# High-speed manufacturing of highly regular femtosecond laser-induced periodic surface structures: physical origin of regularity

**DOI:** 10.1038/s41598-017-08788-z

**Published:** 2017-08-16

**Authors:** Iaroslav Gnilitskyi, Thibault J.-Y. Derrien, Yoann Levy, Nadezhda M. Bulgakova, Tomáš Mocek, Leonardo Orazi

**Affiliations:** 1 DISMI, University of Modena and Reggio Emilia (UNIMORE), 2 via Amendola, Reggio Emilia, 41122 Italy; 2 HiLASE Centre, Institute of Physics AS CR, Za Radnicí 828/5, 25241 Dolní Břežany, Czech Republic

## Abstract

Highly regular laser-induced periodic surface structures (HR-LIPSS) have been fabricated on surfaces of Mo, steel alloy and Ti at a record processing speed on large areas and with a record regularity in the obtained sub-wavelength structures. The physical mechanisms governing LIPSS regularity are identified and linked with the decay length (i.e. the mean free path) of the excited surface electromagnetic waves (SEWs). The dispersion of the LIPSS orientation angle well correlates with the SEWs decay length: the shorter this length, the more regular are the LIPSS. A material dependent criterion for obtaining HR-LIPSS is proposed for a large variety of metallic materials. It has been found that decreasing the spot size close to the SEW decay length is a key for covering several cm^2^ of material surface by HR-LIPSS in a few seconds. Theoretical predictions suggest that reducing the laser wavelength can provide the possibility of HR-LIPSS production on principally any metal. This new achievement in the unprecedented level of control over the laser-induced periodic structure formation makes this laser-writing technology to be flexible, robust and, hence, highly competitive for advanced industrial applications based on surface nanostructuring.

## Introduction

Nanostructured surfaces are of high interest for various scientific, biomedical and industrial applications^1–3^. Numerous methods exist to produce nanostructured surfaces based on sputtering, chemical and plasma etching, chemical vapor deposition, self-assembly, nanoimprint, optical and physical lithography^1–6^. Lithography techniques such as electron- and focused-ion- beam lithographies, and photolithography^1–4, 6^ are most commonly used and provide high-precision nanostructuring. However, the lithography methods are multi-step, employing masks that makes them costly, time-consuming^3, 4^, mainly limited to semiconductor materials, and must be performed under vacuum or specific environment during processing.

Ultrashort laser irradiation is proven as a robust, cheaper alternative to lithography to nano-structure surfaces of various materials via formation of so-called laser-induced periodic surface structures (LIPSS)^7^. The LIPSS represent a periodic surface relief which appears in the two main forms referred as the low spatial frequency LIPSS (LSFL) and the high spatial frequency LIPSS (HSFL) depending on their periodicity^8^. In this work, we limit our study only to the LSFL with periodicity of the order of laser wavelength, which originate from an interference of the incident light with surface electromagnetic wave excited by irradiation^8^. Several application fields have emerged, based on this process, such as modification of surface wetting and tribology properties, surface coloration and marking^9–12^.

Achieving the control over nanostructure formation in these applications remains difficult because of high sensitivity of this process to the type of material and laser irradiation parameters that hinders reproducibility and uniformity of periodic patterns. Although the first observation of LIPSS revealed their good regularity^13^, considerable progress in controlling precision of LIPSS fabrication was made not long ago, though limited to specific materials^14^. More recently, the marking process was distinctly accelerated up to industrial throughputs^15^, opening the way for a faster development of nanostructure-related applications.

So far, the physical origin of the material-dependent regularity remains unclear. Ruiz de la Cruz *et al*.^15^ suggested an electrodynamic mechanism linked with the orientation of laser-beam scanning velocity relative to light polarization. Existing theoretical investigations of the electron dynamics at laser-irradiated materials does not yet include laser scanning^16^ due to complexity of involved interrelated processes, that should be overcome in the incoming years. Large body of investigations reveal that the quality of nanostructures imprinted on surfaces of various kind depends on involved mechanisms and the processes such as surface melting^17^, ablation^7^, and oxidation^18^, all originating from an initial periodic modulation of the deposited electromagnetic energy over the irradiated surface. It has been found that laser-induced oxidation^18^ and partial melting^17^ allow to obtain a very good regularity in the induced pattern in cases of thin-film configurations, typically higher than for LIPSS generated in the ablation regime. Interestingly, adding of only 3% of second harmonic energy to that of the fundamental (800 nm) wavelength can improve nanostructuring quality^19^.

In this paper, we report on the formation of highly regular LIPSS (HR-LIPSS) using laser pulse fluences well above the ablation thresholds (in the ablation regime) on several metals processed in air and at velocities competitive with industrial standards of nano-manufacturing (~1 cm^2^ in 10 s), similar to silicon^20^. A simple methodology is proposed for explaining the regularity of periodic structures on metal/metalized surfaces, which allows to predict whether HR-LIPSS can be formed. The results reveal that only specific metals undergoing high optical losses at the processing wavelength can exhibit regular periodic structures. Metals, which can exhibit excellent LIPSS regularity, are predicted for several wavelengths. It is anticipated that reducing the laser wavelength can enable achieving HR-LIPSS on principally any metal. These results can push high-throughput laser-induced nanostructuring process to a well controllable technology^21^.

## Results

In this Section, the HR-LIPSS generated and the laser parameters used to achieve them are presented. Afterwards, the theoretical analysis explaining the experimental results is made and predictions to other wavelengths and materials are given.

### Highly regular LIPSS formation

The surfaces of 6 different metals, Al, Ti, Cu, Mo, Au and steel, were processed by femtosecond laser irradiations at the wavelength *λ* = 1030 nm at a high laser-beam scanning velocity using a galvoscanner and a translation stage. The details of the experimental setup and samples preparation are described in Materials and Methods. The irradiation parameters are summarized in Table 1.

**Table 1:** Set of parameters used in the present work and in several references where regular LIPSS were obtained.

Materials	Wavelength *λ* (nm)	Pulse duration *τ* (fs)	Energy per pulse *E* (nJ)	*F* ^*^ [J/cm ^2^] (*F*_abs_^†^ [J/cm^2^])	Repetition rate *f* (kHz)	Scanning velocity *v*	Spot diameter *σ* at 1/e^2^ (*μ*m)	Effective pulse number^#^	Overlapping^§^ (%)	Throughput (10^9^ *μ*m ^2^/h)	DLOA *δθ*	LIPSS period Λ (nm)	Reference
Cr	1030	500	1950	0.039 (0.017)	250	1.5 m/s	80	13.3	92.5	9	13.0° ± 1.0°	913 ± 53	Ref. 15.
**Mo**	1030	213	583	0.69 (0.228)	600	1.7 m/s	10.4	3.67	72.8	**21**	**5.3° ± 0.5°**	845 ± 38	**This work**
	800	50	—	0.07 (0.031)	1	0.04 mm/s	~30	~750	99.9	0.0058	8.3° ± 0.5°	589 ± 30	Ref. 24.
**Ti**	1030	213	500	0.59 (0.228)	600	3 m/s	10.4	2.08	51.9	**38**	**9.2° ± 0.5°**	737 ± 26	**This work**
	800	30	—	0.033 (0.013)	1	0.6 mm/s	22	37	97.2	<0.05	8.5° ± 0.5°	~660	Ref. 25.
**Steel**	1030	213	383	0.45 (0.159)	600	3 m/s	10.4	2.08	51.9	**38**	**9.2° ± 0.5°**	901 ± 38	**This work**
	790	30	—	0.055 —	1	5 mm/s	280	56	98.2	<5.1	15.0° ± 1.0°	600 ± 80	Ref. 9
Ni	1026	232	130	0.46 (0.129)	1	0.5 mm/s	6	12	91.7	—	20.0° ± 0.8°	760 ± 120	Ref. 26.
	800	90	—	0.16 (0.051)	1	2.0 mm/s	40	20	95.0	—	14.6° ± 0.5°	~650	Ref. 27.
Al	1030	213	917	1.08 (0.053)	600	3 m/s	10.4	2.08	51.9	—	26.7° ± 0.5°	842 ± 134	This work
Cu	1030	213	1500	1.77 (0.070)	600	3 m/s	10.4	2.08	51.9	—	23.8° ± 0.5°	956 ± 85	This work
Au	1030	213	4080	4.80 (0.101)	600	3 m/s	10.4	2.08	51.9	—	48.8° ± 1.0°	893 ± 160	This work

Throughputs and DLOAs for highly regular LIPSS (HR-LIPSS) fabricated in this work are highlighted by bold typesetting. ^*^ *F* is the average fluence of individual laser pulses and was estimated by the expression *F* = 4*E*/(*πσ* ^2^), where *σ* is the spot diameter at 1/e^2^ of peak intensity. ^†^The absorbed fluence, *F*_abs_ = (1 − *R*)*F*, was obtained using the room temperature reflection coefficient *R* from Johnson *et al*.^28^ for Ti and Cr, from Ordal *et al*.^29^ for Mo, from Palik^30^ for Ni, Al, Cu and Au. For steel, effective medium theory is used (see also Table 2). However, it must be emphasized that depending on metals, the optical properties can significantly vary during the irradiation, leading to a drop in the reflectivity and an increased absorbed fluence^31^. ^#^The pulse number is *N* = *fσ*/*v* where *f* is the repetition rate of the laser and *v* is the scanning velocity. ^§^The overlap is estimated as (1 − 1/*N*) × 100%.

**Figure 1:**
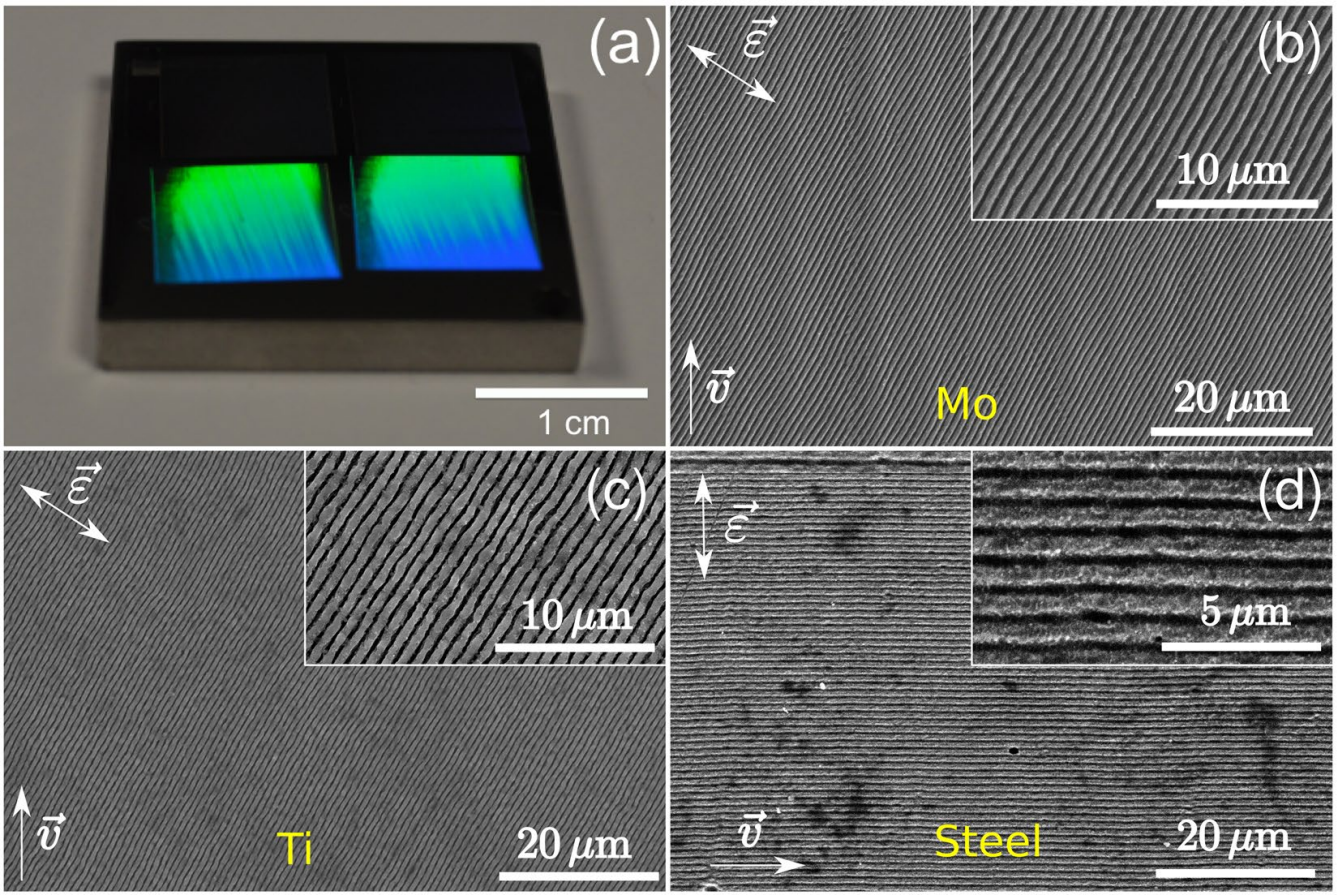
Demonstration of high regularity of LIPSS obtained in this study on several metals. Arrows indicate directions of laser field polarization ($\mathop{ {\mathcal E} }\limits^{\longrightarrow}$) and laser beam scanning ($\mathop{v}\limits^{\longrightarrow}$). (**a**) Human-scale view of a stainless steel AISI 316 L sample covered with HR-LIPSS under ambient light conditions of the laboratory. Color arises from the diffraction of ambient light on the nanostructured material. (**b**–**d**) Respectively Secondary Electron Microscope (SEM) images of the Mo (effective pulse number *N* ~ 3.7, average fluence *F* = 0.69 J/cm^2^), Ti (*N* ~ 2.1, *F* = 0.59 J/cm^2^), and stainless steel (*N* ~ 2.1, *F* = 0.45 J/cm^2^) samples covered with HR-LIPSS. Steel comprises iron with 16.87% Cr and 10.05% Ni. Insets in Figs (**b**–**d**) show magnified views of corresponding images [2× in (**b**) and (**c**), 4× in (**d**)].

For all the irradiated materials, LIPSS with spatial periods close to the laser wavelength *λ* and with orientation perpendicular to the laser light polarization were formed. These structures are known as “Low Spatial Frequency LIPSS” (LSFL)^7, 22^. Among materials studied, HR-LIPSS could only be obtained on Ti, Mo and steel (see Fig. 1). On the rest of the metals surfaces (Au, Cu and Al), we were not able to achieve a high quality of ripples (see images on Fig. S4 in Supplementary Information). In particular, Fig. 1 shows that, for obtaining highly regular structures in our irradiation regimes, scanning velocity has not to be obligatory perpendicular to the polarization direction but can be also up to 45°, in some contradiction to Ruiz de la Cruz *et al*.^15^. To characterize the regularity in a repeatable manner, a systematic approach was used on the basis of an existing measurement procedure^22^. On each SEM picture, the local orientation of the LIPSS was analyzed. From the distribution of the orientation angle, spreading of the latter was extracted which we call the dispersion in the LIPSS orientation angle (DLOA). It is comparable to the angular opening of the two-dimensional Fourier transform (2D-FT) of the original SEM image. However, the technique for determining the DLOA is more robust than an evaluation of the angular diameter which can be approximate on 2D-FT images. This procedure, using an open-source software^23^ based on structure tensor analysis of microscope images, is detailed in Materials and Methods and was systematically applied to SEM images of processed surfaces. The results of the DLOA measurements are summarized in Table 2.

**Table 2:** Properties of excitable SPPs at several air-metal interfaces upon laser irradiation at *λ* = 1030 nm.

Interface	${\rm{\Re }}{\bf{e}}{\boldsymbol{(}}{\varepsilon }_{{\rm{s}}{\rm{a}}{\rm{m}}{\rm{p}}{\rm{l}}{\rm{e}}}{\boldsymbol{)}}$	ℑ*m*(*ε*_sample_)	Optical data	*L*_SPP_, *μ*m	DLOA *δθ*	Exp. reference
air/Cr	−0.63	24.92	Ref. 28.	4.1	13.0° ± 1.0°	Ref. 15.
**air/Mo**	−11.73	20.30	Ref. 29.	**4.3**	**5.3° ± 0.5°**	**This work**
**air/Ti**	−4.27	27.28	Ref. 28.	**4.6**	**9.2° ± 0.5°**	**This work**
**air/steel**	−7.98	28.76	E.M.T.	**5.0**	**9.2° ± 0.5°**	**This work**
air/Ni	−17.81	28.97	Ref. 30.	6.4	20.0° ± 0.8°	Ref. 26.
air/Al	−97.59	25.27	Ref. 30.	65.0	26.7° ± 0.5°	This work
air/Cu	−46.60	4.72	Ref. 30.	73.8	23.8° ± 0.5°	This work
air/Au	−49.59	3.81	Ref. 30.	103.1	48.8° ± 1.0°	This work

DLOA *δθ* determination is detailed in Materials and Methods. E.M.T. denotes the effective medium theory (the Lorentz-Lorenz formula^49^ was used to calculate the dielectric permittivity of steel as function of the volume fraction of Fe, Ni and Cr contained in the sample). The parameters of HR-LIPSS fabricated in this work are highlighted as in Table 1.

The nanostructures on Mo exhibit the highest regularity, to our knowledge, obtained to the date in the ablation regime, with the DLOA *δθ* smaller than ~6°. No visible bifurcation points are seen on the processed Mo surface. So far, the best quality LIPSS, which we could find and analyze from the literature (at a similar wavelength and similar throughput), were obtained on Cr surface by Ruiz de la Cruz *et al*.^15^. They exhibit the DLOA *δθ* of 13.0° ± 1.0° according to the measurement protocol applied for this study (see details in Materials and Methods, *Measurements of the regularity*). It must be noted, however, that the cited image^15^ has other magnification and number of LIPSS available for measurement, which can have an influence on the measurement protocol as shown in Materials and Methods.

Although showing very few bifurcation points on comparable areas, the highly regular structures generated on Ti and steel [Fig. 1(c) and (d) respectively] exhibit DLOA below 10°. The series of experiments carried out in this study on the different metallic sample surfaces are compared with available data found in the literature as summarized in Table 1. Although DLOA variations in different works could be attributed to the use of a specific range of laser fluence for each material (see Table 1), we propose an energy-independent model to explain the periodic structure regularity for the studied materials based on their optical properties at room temperature.

Among six irradiated materials, the highest regularity was obtained on Mo with laser polarization oriented at ~45° relative to the scanning direction. Hence, to achieve the best LIPSS quality, the laser polarization direction should not be strictly perpendicular to the laser scanning direction. However, similarly to^15^, the poorest quality of periodic structures was obtained when the scanning and polarization directions are aligned. The effect of alignment/misalignment of scanning and polarization directions calls for further studies, which are now under progress. Below we propose a simple model and consider a general case of single spot irradiation to address the question on the physical origin of the high regularity of the LIPSS.

### High areal-throughput nanostructuring

Using a high repetition rate laser (see the experimental protocol described in Materials and Methods), large surface area ${\mathscr{A}}$ can be irradiated in a short time Δ*t* at scanning velocities *v* = 1–3 m/s, depending on the overlap between irradiation spots (see Table 1). This defines the areal-throughput $\eta [{\mu }{{\rm{m}}}^{{\rm{2}}}/{\rm{h}}]=3.6\times {10}^{15}{\mathscr{A}}[{{\rm{m}}}^{2}]/{\rm{\Delta }}t[{\rm{s}}]$. The values of 2.1 × 10^10^ *μ*m^2^/h for Mo and of 3.8 × 10^10^ *μ*m^2^/h for Ti and steel were achieved, which are only one order of magnitude below those achieved in traditional lithography techniques, while with the advantage of single step processing. This comparison is added to Fig. S1 adapted from ref. 4 (see Supplementary Information) where the existing techniques of surface nano-manufacturing and their respective areal-throughputs are summarized. Although a comparable throughput was previously reported^15^, our structures exhibit higher regularity at higher throughput for several metals. In particular, the main new features introduced in our experiments are using a small laser spot size, reduced to 10.4 *μ*m (FWHM), against 80 *μ*m (FWHM) in ref. 15 and considerably reduced overlapping between subsequent pulses and scanning lines as compared to other studies. It should be mentioned that further decreasing of the overlap between irradiation spots leads to a decrease of the regularity as the overlapping of the laser field with the pattern created by previous pulses replicates the latter to the new irradiated area as explained by Öktem *et al*.^18^.

### Origin of LIPSS regularity

Recently, He *et al*.^32^ proposed to analyze the regularity of LIPSS by using the inverse Fourier transformation for converting the Sipe efficacy factor calculated along with the transient excitation of Si (the so-called “Drude-Sipe” model^33–35^) from the wave vector domain to the real spatial domain. Based on this approach, the model allowed to reveal the presence of long-range periodicity structures and bifurcation points, defined as localized geometrical aberrations, which must be suppressed for the development of applications based on highly regular nanostructures. Skolski *et al*.^36–38^ generalized this approach using numerical simulations of laser pulse scattering on the surface roughness. Such an approach is promising for predictions of bifurcation points emerging at long range, far from the scattering centers formed after the first laser-pulse irradiation. Although the nanoscale topography of the laser-induced roughness is still unpredictable, the role of defects in the formation of LIPSS was well underlined in a series of works^34, 35, 39, 40^.

It must be admitted that LIPSS formation is an intricate process which involves material ablation/relocation happening well after the laser pulse action. However, it is widely accepted that formation mechanism of LSFL is initiated by transient excitation of Surface Electromagnetic Waves (SEW), which, via interference with the incident laser wave, form a periodic pattern of laser energy absorption on the irradiated surface^8, 33, 34, 41–43^, thus creating a modulated temperature distribution^44^. Assuming that LIPSS are formed via the excitation of Surface Plasmon Polaritons (SPPs)^34, 41^, the 1/e^2^-decay length of SPPs (i.e. their mean free path, denoted by *L*_SPP_) can be calculated and associated with the regularity of the obtained periodic structures for several metals. The properties of this particular type of SEW are given by their dispersion relation, which at an air-material interface is expressed as^45, 46^

1 $$\frac{{k}_{{\rm{air}}}}{{\varepsilon }_{{\rm{air}}}}+\frac{{k}_{{\rm{sample}}}}{{\varepsilon }_{{\rm{sample}}}}=0$$

where *ε*_*j*_ is the dielectric permittivity and *k*_*j*_ is the SPP wave vector in the medium *j*. In materials with losses (where imaginary part of the dielectric permittivity *ε*_sample_ is non-zero), SPPs can be excited if they satisfy to the following general condition^46^:

2 $$\Re e({\varepsilon }_{{\rm{air}}})\,\Re e({\varepsilon }_{{\rm{sample}}})+\Im m({\varepsilon }_{{\rm{air}}})\,\Im m({\varepsilon }_{{\rm{sample}}}) < 0.$$

Accounting that *ε*_air_ = 1, this expression reduces to $\Re e({\varepsilon }_{{\rm{sample}}}) < 0$. The SPPs properties along the interface are described by the SPP wave-number *β* given by^45^

3 $$\beta =\pm \frac{\omega }{c}\sqrt{\frac{{\varepsilon }_{{\rm{air}}}{\varepsilon }_{{\rm{sample}}}}{{\varepsilon }_{{\rm{air}}}+{\varepsilon }_{{\rm{sample}}}}},$$

where *ω* is the laser frequency and *c* is the speed of light. Under the assumption that optical properties of metals do not considerably vary during the laser irradiation, the mean free path of SPPs, *L*_SPP_, can be calculated from the SPP wave number as

4 $${L}_{{\rm{SPP}}}=\frac{1}{2\,\Im m(\beta )}\mathrm{.}$$

The spatial period of the modulated electromagnetic field Λ_SPP_ at the material interface originated from the interference of the incident laser light with the excited SPPs fields can be estimated from the real part of the SPP wave number^45–48^:

5 $${{\rm{\Lambda }}}_{{\rm{SPP}}}=\frac{2\pi }{\Re e(\beta )}\mathrm{.}$$

Assuming that this interference conditions a periodic absorption of laser energy, Λ_SPP_ represents an evaluation of the LIPSS periodicity (see more details in Supplementary Information, section S6).

**Figure 2:**
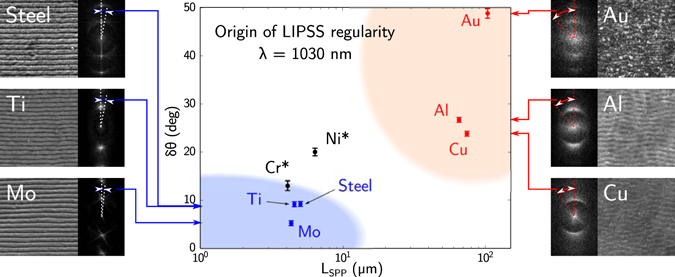
DLOA *δθ* as a function of the calculated mean free path *L*_SPP_ of SEW. SEM images obtained with the same magnification are shown for metals studied in this paper (irradiation conditions are given in Table 1) with the corresponding 2D-FT images. Note that the angular sizes displayed on the 2D-FT are comparable to but not as precise as the DLOA used here. Asterisks stand for DLOA *δθ* data for Cr and Ni, estimated from the images of refs 15 and 26 respectively. Vertical error bars were evaluated from the convergence of the DLOA *δθ* (see Materials and Methods). The LIPSS fabricated in this work, which exhibit high and low regularities, are marked respectively by blue and pink. Materials located in the blue-colored area (respectively in pink-colored area) are suitable (respectively non-suitable) for the HR-LIPSS formation.

This simple model allows to get first insight on how the regularity of periodic structures is linked with the mean free path of SPPs excited on surfaces of laser-irradiated metals. The results of calculations, based on Eqs (2) and (4), for the air-metal interfaces of the samples of interest are reported in Table 2 together with the DLOA *δθ* of the formed LIPSS and plotted in Fig. 2. A strong correlation between the SPP mean free path with the DLOA *δθ* estimated from the experimental images is observed. Materials, which exhibit HR-LIPSS, have SPP mean free paths smaller than ~15 *μ*m at the given irradiation wavelength as evaluated theoretically. Furthermore, the highest regularities in the obtained LIPSS patterns are exhibited by materials with high optical damping (with a high imaginary part compared to the absolute value of the real part in their dielectric permittivity).

**Figure 3:**
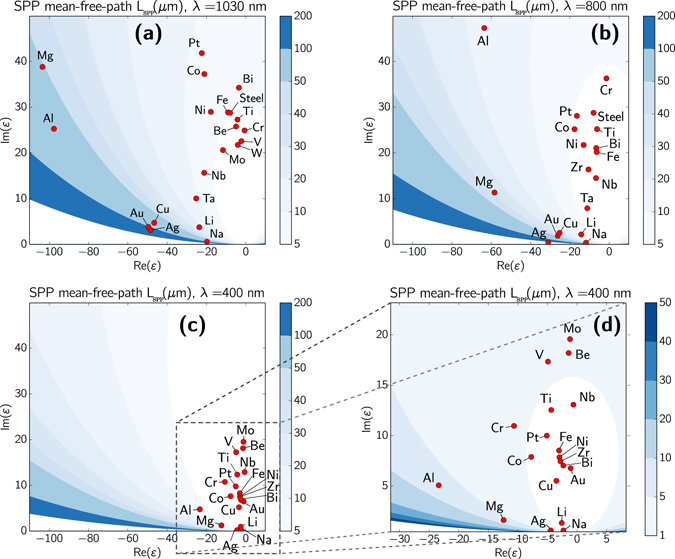
SPP mean free path *L*_SPP_ for different metals at three laser wavelengths *λ* = 1030 nm (**a**), 800 nm (**b**) and 400 nm (**c**,**d**). According to our predictions, metals with small *L*_SPP_ (located in the bright regions) are suitable for HR-LIPSS generation at the corresponding laser wavelengths. Color scales are given in micrometers.

Figure 3(a) presents the *L*_SPP_ values as a function of the real and imaginary parts of the dielectric permittivity, evaluated by Eq. (4) for more than 20 metals at *λ* = 1030 nm, assuming no change of the refractive index during laser irradiation. Two groups of materials can be identified: (i) a group of materials with high optical damping and the SPP decay lengths in the range of 1 to 20 *μ*m, which includes all metals from Table 1 that exhibit HR-LIPSS, and (ii) a group consisting of Al, Mg, Cu, Ag and Au with the SPP decay lengths larger than 50 *μ*m. From comparison of Figs 2 and 3(a), the direct correlation is observed between material damping properties and LIPSS regularity for the materials analyzed in this paper. We underline that metals of the first group with high optical damping and small SPP decay length demonstrate in experiments the high regularity LIPSS formation (to determine the optical properties of steel, we applied the Lorentz-Lorenz formula of effective medium^49^) whereas metals of the second group exhibit low regularity of LIPSS (their SEM images are given in Supplementary Information, Fig. S4).

### Effect of the irradiation spot size

By comparing *L*_SPP_ with the typical spot size used in this work (*σ* = 10.4 *μ*m), we have found that materials for which ${L}_{{\rm{SPP}}}\lesssim \sigma$ exhibit an excellent LIPSS regularity (small DLOA *δθ*) while materials for which ${L}_{{\rm{SPP}}}\gg \sigma$ show poor regularity (relatively large DLOA *δθ*). Irradiation spot size was recently identified as being an important parameter for achieving an excellent regularity of nanostructure formation in thin film oxidation regimes^18^. It was suggested that the reduced size of the irradiation spot secures that all points under the beam contribute to the mutual electromagnetic field, thus avoiding independently created structures. Thus, according to ref. 18, the spatial coherence of the excited surface waves can be preserved more easily on a small irradiation area. Note that the initial observation of a coherent link between overlapping irradiation spots was proposed by *Fauchet and Siegman* ^8, 50^.

The following explanation for the preservation of coherence of SEWs (SPP) generated by the laser pulse can be proposed. The SEWs can be initiated by any sub-wavelength scattering centers^39, 51^ such as point defects, dipole-like nanohumps forming the sample roughness, nanoparticles or even scratches present on the surface^52–54^. When propagating to a large distance and experiencing interactions with numerous scattering centers, SEWs are loosing their initial coherence properties. Contrary to ref. 18, we hypothesize that the excited SEWs remain more *independent* (when their mean free path is small) within the irradiation spot, thus preserving their initial coherence with incoming laser light for forming *a periodic absorption pattern*. This is also in line with our observations that metals with short SPP decay lengths allow for better periodicity compared to metals with long SPP decay lengths. Interaction between rapidly decaying SEWs is reduced that enables to support their coherence with the incoming light even within relatively large irradiation spots^15^.

### Predictions of materials suitable for Highly Regular LIPSS formation

The proposed above methodology, which is based the on *L*_SPP_ evaluation, allows to select the materials exhibiting high optical damping and hence suitable for HR-LIPSS inscription at particular irradiation conditions. The results of calculations of *L*_SPP_ as a function of the real and imaginary parts of the dielectric functions are shown in Fig. 3(a–c) for three laser wavelengths, *λ* = 1030, 800, and 400 nm. Interestingly, the number of materials with a short decay length *L*_SPP_, which can enable HR-LIPSS generation, is increasing with decreasing the wavelength. At *λ* = 1030 nm, besides Mo, Ti, Ni, Cr, and steel, such metals as Nb, W, V, Be, Bi, Co, Pt and pure Fe are identified as candidates for HR-LIPSS formation while Ag falls to the group of low regularity LIPSS together with Au, Al, and Cu whose SEM images with LIPSS are shown in Supplementary Information, Fig. S4, and additionally with Mg, Na, Li, and Ta. At *λ* = 800 nm, the group of the HR-LIPSS metals is enriched with Li, Ta, Zr, and Bi. With further decreasing laser wavelength to *λ* = 400 nm, our theory predicts that the majority of analyzed metals can enable HR-LIPSS formation, including gold and copper^55^ for which a high regularity of LIPSS is hardly achievable at longer wavelengths. As a whole, we predict that, using blue or near-UV laser light, regularity of the laser-induced periodic structures can be dramatically improved for many metals. This can open extraordinary opportunities for extending our experimental results to a wide range of materials and enable the development of high-quality demanding applications (optical gratings as an example). For the calculations whose results are presented in Fig. 3, the data on dielectric permittivities were taken from the previous multi-material study^46^. The corresponding code is available online^56^ to help the community to compare their future works with our predictions (see the “Code availability” subsection in Materials and Methods).

## Discussion

The above predictions are based on optical properties of metals (*n* and *k*, or equivalently *ε*) at the room temperature under equilibrium conditions. The data on refractive indices and extinction coefficients published in different literature sources for same materials can noticeably vary. These variations can produce uncertainty in the evaluated data with consequences for the accuracy of our predictions. Calculations however show that varying the real and imaginary parts of the dielectric permittivity does not strongly affect the predicted LIPSS periodicity, Λ_SPP_ (see Supplementary Information, Figs S6 and S7). The Λ_SPP_ and *δ*Λ_SPP_ values were estimated using analytic expressions given in Supplementary Information (section S6). One can expect a stronger effect on the SPP decay length from swift non-equilibrium heating of the electron subsystem in metals by ultrashort laser pulses and associated dynamic change of the dielectric permittivity^31^.

At relatively high laser fluences which are exploited in this work for generation of LIPSS on metal surfaces, free electrons can gain energies of several electron-Volts during the laser pulse action^31, 57^. As a consequence, their optical response can be dramatically changing, thus affecting the results of our predictions. Hence, it is important to investigate the possible changes in the SPP mean free path during laser irradiation.

**Figure 4:**
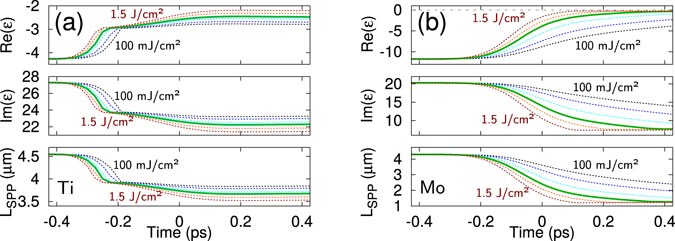
Estimations of dynamic changes of the optical properties upon irradiation of Ti (**a**) and Mo (**b**) by a 213 fs laser pulse at *λ* = 1030 nm. Variations of the real and imaginary parts of the dielectric permittivity *ε* and the associated SPP decay length are shown at different laser fluences (colored lines with increasing fluence from right to left): 0.1, 0.2, 0.4, 0.6, 1.0 and 1.5 J/cm^2^ for titanium and 0.1, 0.2, 0.4, 0.69, 1.0 and 1.5 J/cm^2^ for molybdenum. In each graph, the green solid line corresponds to the experimental fluence (averaged over the irradiation spot) at which the reported LIPSS were produced.

It has been shown^31^ that, in the case of copper, the real and the imaginary parts of the dielectric permittivity are strongly changing functions of the electron temperature even at temperature levels well below the Fermi one. Following the approach reported in^31^, the temporal variations of the dielectric permittivity were modeled numerically for titanium and molybdenum in the course of femtosecond laser irradiation. For these aims, the two temperature model (TTM)^57^ was supplemented with the computation of the optical properties in the frame of the Drude model. The dielectric permittivity expressed as

6 $$\varepsilon =1-\frac{{\omega }_{pe}^{2}}{{\omega }^{2}+{\nu }_{{\rm{eff}}}^{2}}+i\frac{{\nu }_{{\rm{eff}}}{\omega }_{pe}^{2}}{\omega ({\omega }^{2}+{\nu }_{{\rm{eff}}}^{2})}$$

was implemented in the numerical scheme of the TTM, with *ν*_eff_ to be the temperature dependent effective collision frequency^31^, and *ω* and *ω*_*pe*_ to be respectively the laser and plasma frequency. Details of the modeling can be found in the Supplementary Information, section S5. The calculations thus allow for self-consistent simulations of the temporal evolution of the electron and lattice temperatures and the dielectric permittivity. The latter enables to calculate the temporal variations of *L*_SPP_ and provides the reflection and absorption coefficients, *R*(*t*) and ${\alpha }_{\text{abs}}(z,t)=4\pi \,{\rm{I}}{\rm{m}}(\sqrt{\varepsilon (z,t)})/\lambda$ respectively (*z* is the distance from the sample surface to a point inside the bulk). The results of simulations for Ti and Mo are presented in Fig. 4(a) and (b) respectively, showing the evolution of the dielectric permittivity and the corresponding variations of the SPP mean free path during the laser pulse irradiation at fluences, which are typical for production of the HR-LIPSS reported here. Hence, an important conclusion can be made that *L*_SPP_ is decreasing during the irradiation. This provides vital consequences for the HR-LIPSS direct writing. During the irradiation, materials with initially small *L*_SPP_ processed with ultrashort laser pulses will continue to satisfy the condition for obtaining highly regular periodic structures, as indicated in Table 2. Furthermore, according to our prediction, the regularity can be even improving upon swift laser heating due to dynamically decreasing *L*_SPP_ value. This can imply that some materials, which have relatively large *L*_SPP_ under normal conditions, can exhibit a tendency of improved LIPSS regularity with increasing laser fluence, which calls for further studies.

## Conclusion

In this paper, a very high regularity of near-wavelength periodic patterns was obtained in the laser-ablative regimes on the surfaces of Mo, steel and Ti at a competitive throughput of ~1 cm^2^ per 10 s. A theoretical explanation of the regularity was proposed which was verified by LIPSS formation on surfaces of several metals irradiated under the conditions above the corresponding ablation thresholds. The proposed theory has uncovered an important role of the reduced spot size and its linking with the mean free path of Surface Electromagnetic Waves in triggering the formation of regular structures. It has been found that metals with small SPP mean free paths, of order of several *μ*m, support a good coherence of SPP with the incident laser light, thus enabling the formation of HR-LIPSS. Increasing *L*_SPP_ results in the increased interaction of the excited surface waves, both between them and with various surface defects, which have consequences in decoherence with incoming light and reduction of LIPSS regularity. It was also revealed that, for the group of materials which exhibit small SPP mean free paths, a small irradiation spot, of the order of *L*_SPP_, enhances regularity of the generated periodic structures that can be also explained by reduced interaction of SPPs created on different sites of the spot. Numerical modeling based on the TTM supplemented by the Drude model with electron-temperature-dependent electron collision frequency has demonstrated that, at ultrafast laser heating, *L*_SPP_ of metals is decreasing that can result in a higher regularity of periodic structures.

The proposed model explains well the results of our experiments: high LIPSS regularity on Ti, Mo, and steel surfaces, and the low regularity for Al, Cu, and Au. The model predictability is also supported by the HR-LIPSS obtained on Cr^15^ and Ni^26^. Further predictions have been made for a wide range of metals for several laser wavelengths. It is anticipated that reducing laser wavelength from near-IR light to the spectral range from green to near-UV can enable formation of highly regular sub-wavelength periodic structures on surfaces of numerous metals that requires further studies. As was proven in previous studies^43, 58^, the period of structures can be modified (increased or decreased) by more than 50% via adjusting the angle of incidence and polarization, to be addressed in further developing of the proposed theory. The control over writing laser-induced periodic structures at reduced number of pulses opens new unprecedented opportunities for laser nanoprocessing of large surface areas of materials, suitable for industrial applications^21^.

## Materials and Methods

### Materials preparation

The samples of Ti and Mo consist of 300 nm-thick coatings deposited on glass (quartz) substrates using physical vapor deposition (PVD) by magnetron sputtering a 3-inch Mo and Ti targets (purity for both was 99.95 %) in Ar atmosphere. Before deposition, the chamber was pumped to a base pressure lower than 10^−6^ Torr. During the deposition, the pressure was fixed at 5 · 10^−3^ Torr, and the substrate was rotated to provide a uniform coating thickness. Copper (99.9% purity), aluminum (99.9% purity) and stainless steel (AISI 316 L) samples with dimensions of 25 × 25 × 5 mm were mechanically polished and cleaned in ethanol before irradiation. The commercial Au alloy 18k Yellow Gold (12.5% Silver) samples were plates of 1 mm thickness. Optical data for Au and Ag were taken from ref. 28.

The surface morphology of the patterned samples was investigated by scanning electron microscopy (SEM), imaging with secondary electrons and custom modes using a FEI Nova NanoSEM 450 with Bruker QUANTAX-200 X-EDS.

### Laser irradiation of metals

LIPSS were produced using a commercial Yb-doped solid state laser system PHAROS, providing *τ* = 213 fs (FWHM) pulses at a central wavelength of 1030 nm with a spectral width of 15 nm; M^2^ ≃ 1.1.

The pulse repetition rate was same in all experiments and set to 600 kHz. At this repetition rate the laser system generated up to 20 W of average power. The laser was then coupled with a galvanometer scanning system (ProSeries Cambridge Technology) and focused by an f-theta lens with a focal length of 56 mm. The approximate diameter of the irradiation spot on the sample surfaces was 7.34 μm at 1/e of the peak intensity or, equivalently, 10.4 *μ*m at 1/e^2^. To evaluate the laser beam diameter, we used the Gaussian optics formalism. The positioning of sample surfaces to the geometrical focus of the laser beam was carried out by moving the sample along the beam propagation direction with searching for both the smallest damage spot on the surface and the brightest ablation plasma. The uncertainty of the beam diameter was evaluated through application of the differential error analysis theory where four terms were taken into account, connected with uncertainties of (i) laser wavelength (dispersion), (ii) focal distance of *θ*-lens, (iii) its diameter, and (iv) beam quality factor M^2^. This estimation yields ~0.38 *μ*m.

**Figure 5:**
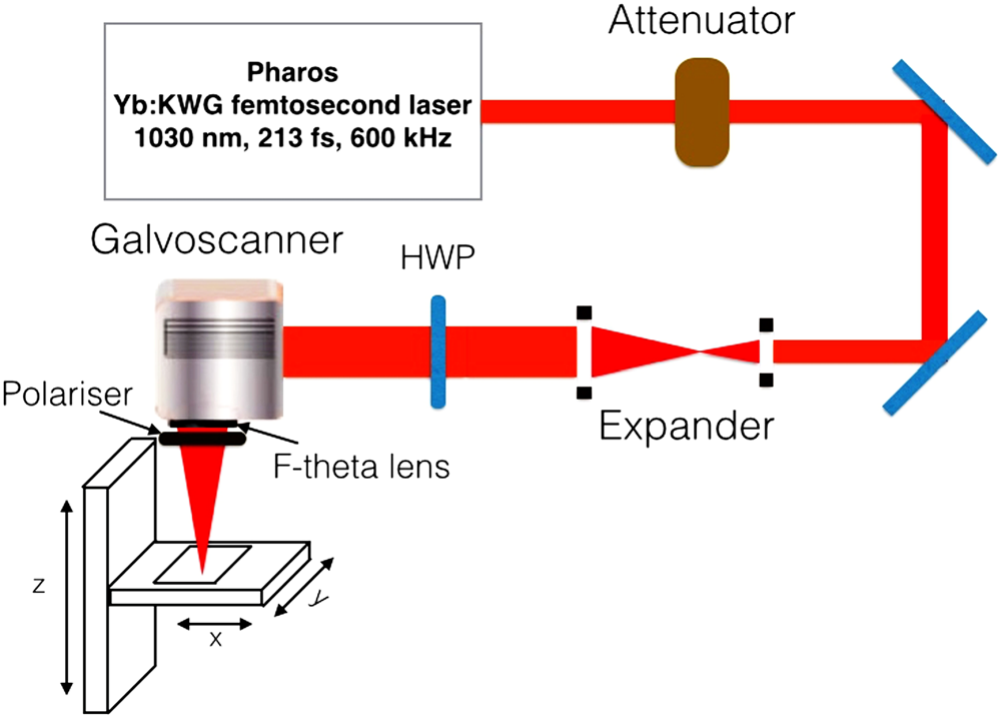
Scheme of the experimental setup used for irradiation of metallic samples. HWP: Half-wave plate.

To limit the average power of laser pulses on the sample surface, a motorized attenuator was utilized to transmit about 2.5% of incident power of the laser system. Transmittance of the focusing system was estimated independently prior to the experiments and was measured to be 80 % at the laser wavelength. The general scheme of the LIPSS generation setup is shown in Fig. 5. We mention that, in our irradiation regime the heat accumulation between pulses can be disregarded, see Supplementary Information.

### Measurements of the regularity

**Figure 6:**
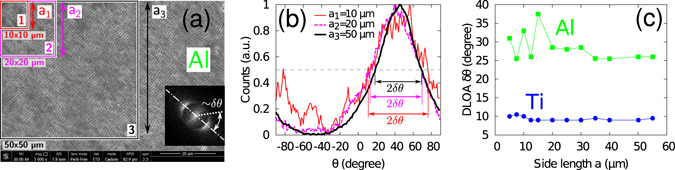
Effect of the measurement area on the DLOA *δθ*. For different sizes of the LIPSS-covered area chosen for analysis (**a**), different spectra (**b**) of orientations *θ* can be obtained with the module *OrientationJ* ^23^ of the open software *ImageJ* ^59^. The normalized spectra of orientation are shown for the case of Al. The orientation axis ranges from −90° to +90° (orientation is *π*-periodic: the orientation angle of +91° corresponds to the orientation of −89°). Note that on (**b**) the distribution is centered around 45°, in line with the LIPSS orientation on (**a**). Colors of lines in (**b**) correspond to areas in (**a**) highlighted by the same colors. (**c**) Good convergence of the DLOA measurements is achieved when the surface area selected for analysis is large enough. Side length *a* refers to the square area (of side *a*_*i*_, *i* = 1, 2, 3) in Fig. (**a**) The measurement performed on Ti is also reported for comparison.

From SEM images, the regularity was analysed by measuring the dispersion of the LIPSS orientation angle, DLOA, *δθ*. A systematic, repeatable procedure was implemented and applied to all laser-processed samples as summarized by Fig. 6.

- We used the freely available *OrientationJ* plugin^23^ written for the *ImageJ* open-source software^59^ based on the tensor structure analysis of the image to be processed. The specific module *Orientation Distribution* with the *Riesz Filters* structure tensor was used. No Gaussian smearing was applied. For each analysis, the angle distribution was corrected from its offset (minimum value of the spectrum) and the half width at half maximum was extracted to obtain the DLOA, *δθ*.
- The magnification of the original SEM images was kept the same, 5,000×, in all measurements in order to keep similar measurement conditions for all samples.
- Orientation distribution analysis was performed over large squared areas to allow for sufficient statistics and the corresponding stabilization of the DLOA value with increasing the size of the analyzed area. Figure 6 shows the analysis procedure and demonstrates stabilization DLOA for Al and Ti. Note that, for Al, the distribution of angles globally narrows as the size of the analyzed surface increases.
- The errors of the DLOA measurements were estimated from the accuracy of the distribution (∼±1°) and from the residual fluctuations in the stabilized area.

### Code availability

The Python code used to produce the data presented in Fig. 3 (manuscript), and S2, S3, S6, S7 from Supplementary Information is available at the following web address: http://www.quantumlap.eu/. Simple access to the code is possible on requests to the corresponding author.

## Electronic supplementary material

Supplementary Information
