# Supplementary material for: High-speed manufacturing of highly regular femtosecond laser-induced periodic surface structures: physical origin of regularity
Source: Sci Rep. 2017 Aug 16;7:8485. doi: 10.1038/s41598-017-08788-z (PMC5559557; doi:10.1038/s41598-017-08788-z)
Supplement: Supplementary file 1 — Supplementary Information [file 41598_2017_8788_MOESM1_ESM.pdf]

High-speed manufacturing of highly regular femtosecond laser-induced periodic surface structures: physical origin of regularity

Iaroslav Gnilitskyi[1,+], Thibault J.-Y. Derrien[2,*,+], Yoann Levy[2], Nadezhda M. Bulgakova[2], Tomáš Mocek[2], and Leonardo Orazi[1]

[1]DISMI, University of Modena and Reggio Emilia (UNIMORE), 2 via Amendola, Reggio Emilia 41122, Italy

[2]HiLASE Centre, Institute of Physics AS CR, Za Radnicí 828/5, 25241 Dolní Břežany, Czech Republic

[*]Corresponding author, e-mail: derrien@fzu.cz

[+]These authors contributed equally to this work

Supplementary Information Contents

S1 High throughput . . . 1

S2 Effect of laser wavelength for obtaining HR-LIPSS . . . 1

S3 Non-regular LIPSS on Au, Al and Cu . . . 3

S4 Scheme of principle of HR-LIPSS writing . . . 4

S5 Modeling of the change in the optical properties . . . 5

S6 Sensitivity of LIPSS period to variations of optical properties . . . 7

S7 Optical model of metallic films . . . 10

S8 Estimations of heat accumulation under the LIPSS formation on Ti and Mo films . . . 11

S9 Effect of surface roughness . . . 12

S10 Acknowledgment . . . 12

References . . . 12

S1 High throughput

The Highly Regular Laser Induced Periodic Surface Structures (HR-LIPSS) reported in this study on Ti, Mo and stainless steel have been processed at areal throughput comparable to recent existing lithography techniques (see Fig. S1, adapted from Ref.[1]). Red ellipse indicates length scales from 100 nm to 1 μm at the processing speed announced in core of the paper.

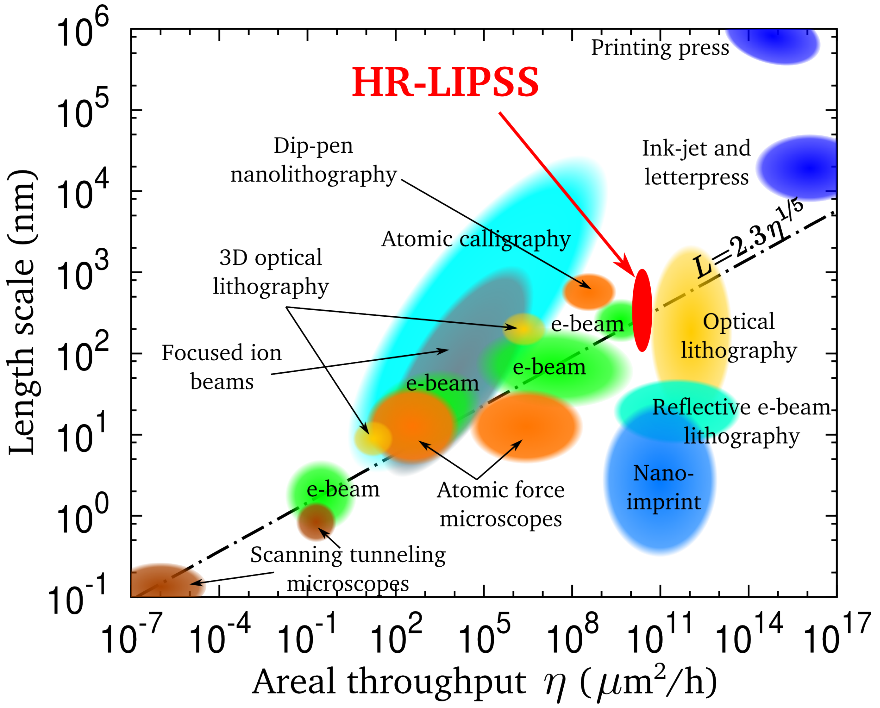

Figure S1. Summary of the nano-manufacturing techniques, adapted from Imboden et al.[1] with some modification and addition of the data of our developed method of surface nanostructuring throughput. The black dot-dashed line stands for Tennant's law.[1] The red ellipse corresponds to the processing method developed in the present work. The length scale of the achievable LIPSS periods is determined by the variations of the laser angle of incidence on the surface. Adapted from Imboden *et al*, Physics Today **67**, 45-50 (2014) [`http://dx.doi.org/10.1063/PT.3.2621`], with the permission of the American Institute of Physics.

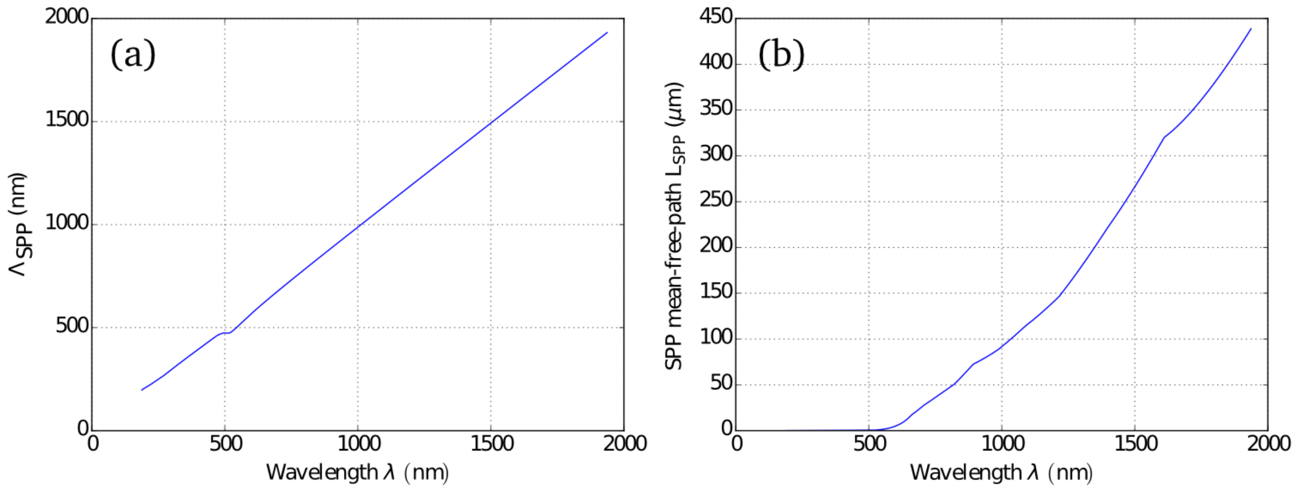

Figure S2. Period (a) and mean free path (b) of SPPs as a function of laser wavelength for Au. Our analysis predicts that HR-LIPSS can be obtained by using a wavelength $\lesssim$ 600 nm.

S2 Effect of laser wavelength for obtaining HR-LIPSS

As seen in Fig. 2 of the main manuscript, the regularity of the LIPSS obtained on Au with 1030-nm wavelength was particularly poor. Using Eqs. (3)-(4) of the manuscript, one can calculate the SPP mean free path L_{SPP} as a function of laser wavelength. The calculation data presented in Fig. S2(b) suggest that it can be possible to write highly regular LIPSS on Au surfaces by using laser wavelengths $\lesssim$ 600 nm. As discussed in the manuscript, the irradiation spot size of several micrometers can facilitate HR-LIPSS formation. The period of the LIPSS is linked to the used wavelength, see Fig. S2(a), where the SPP period that determines the LIPSS period is given for the normal incidence of the laser beam on Au surface. As demonstrated in previous studies, the period can be varied by increasing the angle of laser incidence.[2,3]

Similar calculations performed for Ti predict that highly regular structures can be obtained in a wide range of laser wavelengths, plausibly from UV to ~2 μm (see Fig. S3). In the case of Ti, note that the period prediction shown in Fig. S3(a) does not account for the presence of any oxide layer on the surface, which can strongly affect the obtained result.[4,5]

The proposed prediction model can be applied to a wide range of materials,[6] allowing the control over HR-LIPSS production.

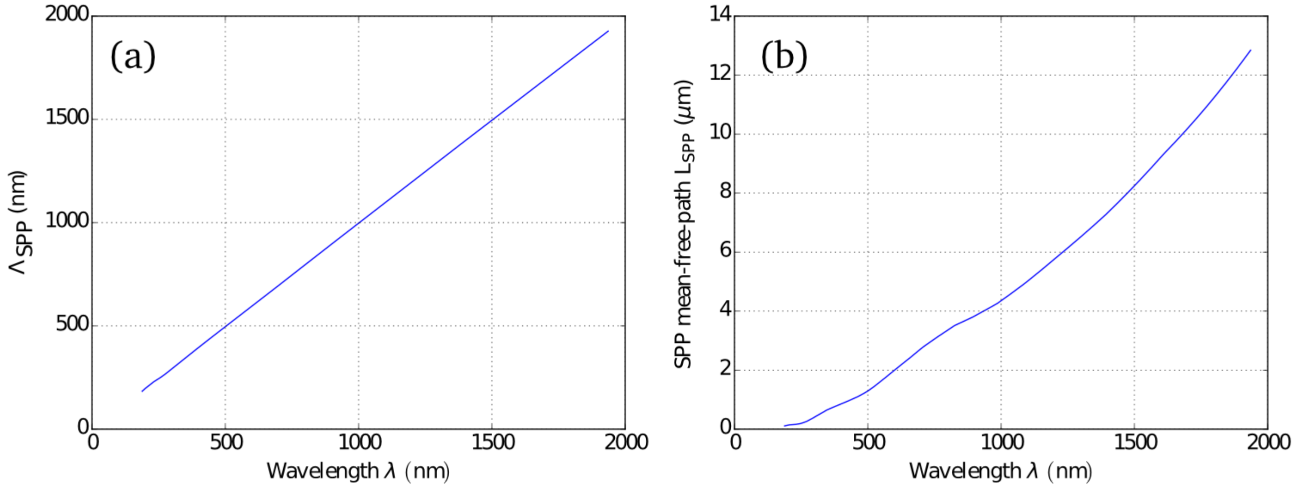

Figure S3. Period (a) and mean free path (b) of SPPs as a function of laser wavelength for Ti. The model predicts high regularity of LIPSS on Ti surfaces even at wavelengths toward mid-IR spectral range.

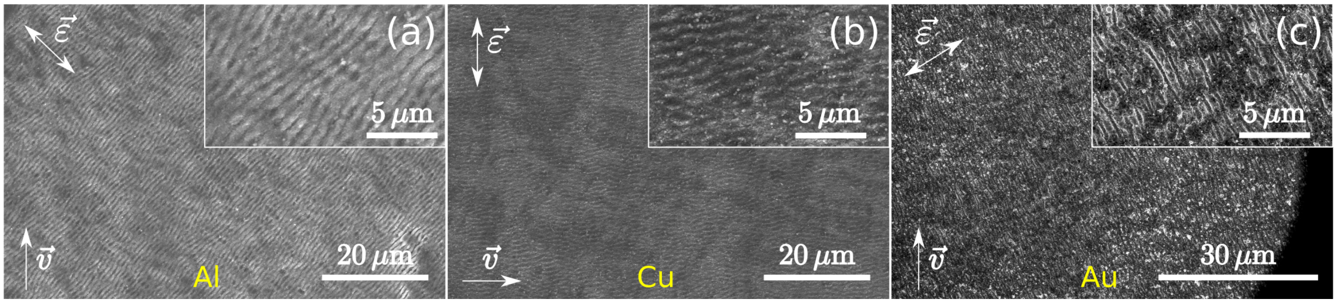

Figure S4. Set of SEM images of Al (effective pulse number $N = 2.08$, average fluence $F = 1.08\,\mathrm{J/cm^2}$), Cu ($N = 2.08$, $F = 1.77\,\mathrm{J/cm^2}$) and Au ($N = 2.08$, $F = 4.80\,\mathrm{J/cm^2}$) exhibiting a low regularity of LIPSS at laser wavelengths of 1030 nm. For each material, the laser beam scanning direction ($\vec{v}$) is indicated relative to the laser polarization ($\vec{\mathscr{E}}$).

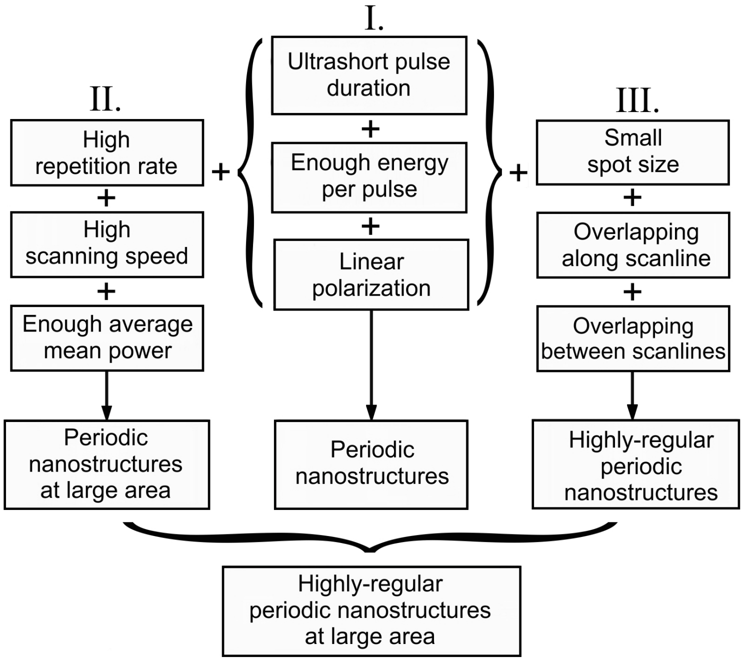

Figure S5. Scheme of principle to obtain HR-LIPSS on large surface area at high speed.

S3 Non-regular LIPSS on Au, Al and Cu

Several types of metals have been irradiated using the protocol described in Materials and Methods of the manuscript. Figure S4 shows LIPSS of low regularity obtained on Al, Cu and Au surfaces (under the same irradiation conditions as on surfaces of Mo, Ti and stainless steel where HR-LIPSS were produced). These results suggest that the LIPSS regularity depends on the irradiated material properties. Periodicities of the LIPSS for these materials were also comparable to the laser wavelength, as given in Table 1 of the manuscript. Note that the angle between beam scanning direction and laser light polarization does not noticeably affect the LIPSS regularity on surfaces of these materials. For these three metals, the SPP model, based on the mean free path L_{SPP} of surface plasmon polaritons, has predicted that DLOA must be large (see Fig. 2 of the manuscript), in agreement with observations.

S4 Scheme of principle of HR-LIPSS writing

Figure S5 summarizes necessary steps for obtaining HR-LIPSS over large areas and at high speed. Basically, to produce periodic nanostructures, multiple irradiation of the surface by polarized laser beam of ultrashort (fs/ps) duration is needed (I). To translate the structures on large surface area at high throughput, high repetition rate and high scanning speed of the beam are needed with enough average mean power

to induce meting/ablation (II). According to our findings, to write highly regular LIPSS, a small spot size with specific overlapping between irradiation spots in scanline and between scanlines is the main requirement (III). I, II, and III together present the way to produce highly regular LIPSS on large area at high production speed, providing that material optical properties enable high regularity of structures, see the manuscript (Fig. 3 and associated text).

S5 Modeling of the change in the optical properties

In this Section we present the details of the modeling of the dynamic variations of metal optical properties induced by the action of ultrashort laser pulses. The results of the modeling are used in the main manuscript for discussing the laser-induced variations of the SPP decay length.

Upon irradiation by high-intensity laser pulses, the optical properties of metals can experience swift variations induced by rapid heating of free electrons to a high temperature, T_e. Therefore, to adequately describe the absorbed laser energy and heating dynamics, the dynamic change of the optical properties has to be calculated together with the balance of laser energy released in the target. The latter is modeled by the two-temperature model[7,8] (TTM) in two dimensions (2D) (along the surface, x, and toward the bulk, z; the surface of the material is located in $z = 0$). The electron subsystem absorbs the laser energy and transfers it to the lattice subsystem (with a temperature T_l) through electron-phonon collisions, modeled by the coupling factor γ:

$$\begin{cases} C_e \dfrac{\partial T_e}{\partial t} = \nabla\left(\kappa_e \nabla T_e\right) - \gamma\left(T_e - T_l\right) + S \\ C_l \dfrac{\partial T_l}{\partial t} = +\gamma(T_e - T_l) \end{cases} \tag{1}$$

In the modeling, the thermal conductivity and the heat capacity of the electron subsystem were considered as linear functions of the electron temperature,[9] $\kappa_e = \kappa_{e,0} T_e / T_l$ and $C_e = A_e T_e$ respectively, while the electron-phonon coupling factor γ and the heat capacity of the lattice C_l were assumed constant. For titanium,[2,10,11] $\gamma = 1.85 \times 10^{18}\,\mathrm{W}/(\mathrm{m}^3 \cdot \mathrm{K})$, $A_e = 329\,\mathrm{J}/(\mathrm{m}^3 \cdot \mathrm{K}^2)$, $\kappa_{e,0} = 22\,\mathrm{W}/(\mathrm{m} \cdot \mathrm{K})$ and $C_l = 2.35 \times 10^6\,\mathrm{J}/(\mathrm{m}^3 \cdot \mathrm{K})$ and for molybdenum[9], $A_e = 350\,\mathrm{J}/(\mathrm{m}^3 \cdot \mathrm{K}^2)$, $\kappa_{e,0} = 135\,\mathrm{W}/(\mathrm{m} \cdot \mathrm{K})$, $\gamma = 1.3 \times 10^{17}\,\mathrm{W}/(\mathrm{m}^3 \cdot \mathrm{K})$ and $C_l = 2.8 \times 10^6\,\mathrm{J}/(\mathrm{m}^3 \cdot \mathrm{K})$. Note that the lattice heat conductivity is negligible at the timescale of interest. The laser source term S depends on the reflection and absorption coefficients,

$R(x,t)$ and $\alpha_{\text{abs}}(x,z,t) = 4\pi\,\text{Im}\left(\sqrt{\varepsilon(x,z,t)}\right)/\lambda$ respectively:

$$S(x,z,t) = (1 - R(x,t))\,\alpha_{\text{abs}}(x,z,t)\,\text{I}_0(x,t)\exp\left(-\int_0^z \alpha_{\text{abs}}(x,z',t)\,\text{d}z'\right).$$

The intensity of the laser beam I_0 incident on the smooth sample surface follows basic Gaussian temporal and spatial profiles corresponding to the experimental conditions: pulse duration of $\tau = 213\,\text{fs}$ (full width at half maximum) and the beam waist $w_0 = \sigma/2 = 5.2\,\mu\text{m}$.

The optical properties are determined by the dielectric permittivity ε, expressed by

$$\varepsilon = 1 - \frac{\omega_{pe}^2}{\omega^2 + \nu_{\text{eff}}^2} + i\frac{\nu_{\text{eff}}\omega_{pe}^2}{\omega\left(\omega^2 + \nu_{\text{eff}}^2\right)} \tag{2}$$

where ω is the laser frequency, $\nu_{\text{eff}} = \min(\nu_e;\nu_c)$ is the effective collision frequency,[12] ν_e is the electron collision frequency and ν_c the critical collision frequency based on the interatomic distance and the electron velocity $\nu_c = (4\pi n_0/3)^{1/3}\sqrt{v_F^2 + k_B T_e/m_e}$ (n_0 is the atomic density and v_F is the Fermi velocity). The electron collision frequency is considered as the sum of the electron-electron and of the electron-ion collisions: $\nu_e = \nu_{ee} + \nu_{ei} = AT_e^2 + BT_l$. The first coefficient A can be estimated from the expression for the electron-electron collision frequency[13] by assuming that the electrons are thermalized to the Fermi distribution so that $E - E_F \approx k_B T_e$. To fit the optical properties to the literature data at room temperature, T_0, within the Drude model (Eq. (2)), the plasma frequency is calculated as $\omega_{pe} = \omega\sqrt{1 - \varepsilon'(T_0) + \varepsilon''^2(T_0)/(1 - \varepsilon'(T_0))}$ and one obtains for titanium $A = 2.2\times10^6\,\text{K}^{-2}\cdot\text{s}^{-1}$. The coefficient B can be deduced from this latter value and from the collision frequency at room temperature T_0, $\nu_{\text{eff}}^{\text{opt}} = \omega\varepsilon''(T_0)/(1 - \varepsilon'(T_0))$, based on the optical properties of metal at thermodynamic equilibrium: $B = \nu_{\text{eff}}^{\text{opt}}/T_0 - AT_0$. From the optical properties of titanium at room temperature[14] (see Table 1), we obtain $B = 3.2\times10^{13}\,\text{K}^{-1}\cdot\text{s}^{-1}$. For molybdenum, similar formalism yields $A = 2.6\times10^6\,\text{K}^{-2}\cdot\text{s}^{-1}$ and $B = 9.8\times10^{12}\,\text{K}^{-1}\cdot\text{s}^{-1}$. The Table 1 summarizes the parameters of the simulations presented in the main manuscript for Mo and Ti.

The numerical code used for solving the combined problem of the energy balance and the optical properties has been developed using a finite differences method[15]. Equations (1) and (2) are solved at each iteration step. The simulation domain consists of a rectangular area $0 \le z \le D$ and $0 \le x \le L$ in 2D cylindrical geometry with $x = 0$ at the irradiation spot center where D is taken large enough to avoid any reflection artifact. The reflective boundary conditions were applied on each boundary. The dimension of

Parameter (unit)	Ti	Ref.	Mo	Ref.
Coefficient κ_{e0} in κ_e ($\mathrm{W \cdot m^{-1} \cdot ^{-1}}$)	22	Ref.[2]	135	Ref.[9]
Coefficient A_e in C_e ($\mathrm{J \cdot m^{-3} \cdot K^{-2}}$)	329	Ref.[2]	350	Ref.[9]
Coupling factor γ ($\mathrm{W\,m^{-3}\,K^{-1}}$)	1.85×10^{18}	Ref.[10]	1.30×10^{17}	Ref.[9]
Lattice heat capacity C_l ($\mathrm{J \cdot m^{-3} \cdot K^{-1}}$)	2.35×10^6	Ref.[2]	2.80×10^6	Ref.[9]
Re(ε) at 300 K	-4.27	Ref.[14]	-11.73	Ref.[16]
Im(ε) at 300 K	27.28	Ref.[14]	20.30	Ref.[16]
Parameter A in ν_{eff} ($\mathrm{K^{-2}\,s^{-2}}$)	2.2×10^6	Deduced	3.2×10^{13}	Deduced
Parameter B in ν_{eff} ($\mathrm{K^{-1}\,s^{-2}}$)	2.6×10^6	Deduced	9.8×10^{12}	Deduced
Atomic density of the material n_0 ($\mathrm{m^{-3}}$)	5.7×10^{28}	Ref.[2]	6.4×10^{28}	Ref.[2]
Fermi energy of the material E_F (eV)	8.84	Ref.[17]	6.2	Ref.[18]

Table 1. Parameters used in the modeling of the change in the optical properties of Mo and Ti upon irradiation by a 213 fs, 1030 nm laser pulse.

the transverse size L was twice the experimental Gaussian radius ($\sigma/2$) to limit the truncation inaccuracy in the laser pulse energy to less than 1% while keeping the computing resources reasonable. The initial temperatures are set to $T_e = T_l = T_0 = 300\,\mathrm{K}$ and the initial optical properties to those of the corresponding temperature (see Table 1).

S6 Sensitivity of LIPSS period to variations of optical properties

The periodicity of LIPSS is determined by the spatial period of the modulated electromagnetic field Λ_{SPP} at the material interface originated from the interference of the incident laser light with the excited SPPs fields.[19] As discussed in the manuscript, the period can be affected not only by the change of the angle of incidence of the laser beam but also by swift heating of the electron subsystem and associated change of optical properties. As a result, the period of the light absorption can transiently be changing, thus influencing the final LIPSS inscribed on the surface.

When it is not possible to perform advanced numerical simulation of the transient refractive index of material during the irradiation, we propose in this section a simplified model based on the uncertainty of the dielectric permittivity ε to calculate on how much the transient periodic pattern of light absorption can be modified during the laser pulse. As an example, for the laser wavelengths of 1030 nm and 800 nm, specific groups of metals should exhibit only small Λ_{SPP} changes of the period during the irradiation (see Figs. S6 and S7 respectively). For 800 nm wavelength, the periods of SPPs excited on Na, Li, Ta, and, to some extent, on Nb are expected, according to our predictions, to be very sensitive to the change of dielectric permittivity upon swift heating of free electrons as discussed in the manuscript. On the contrary,

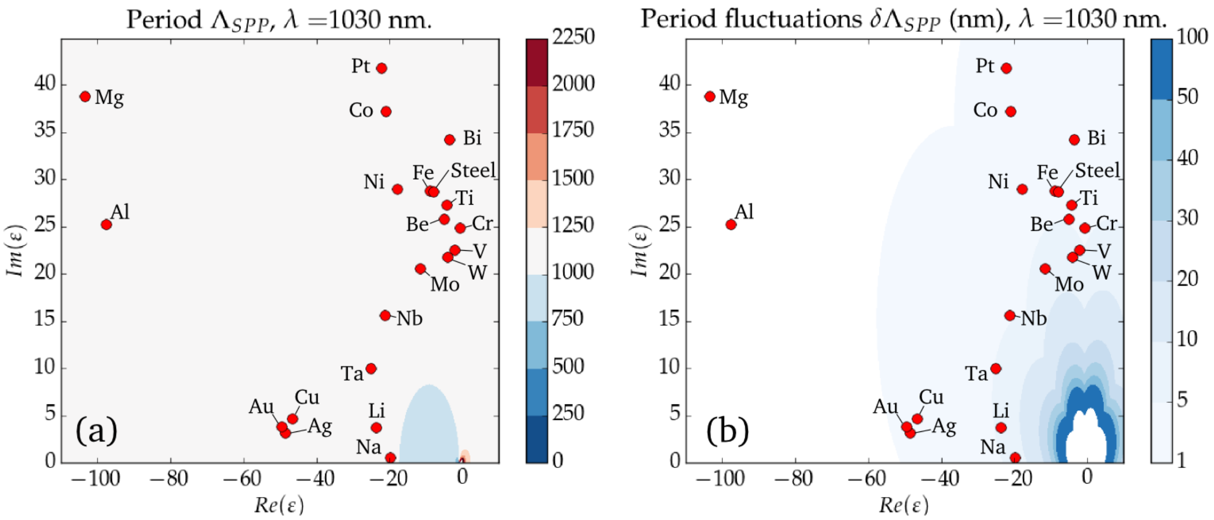

Figure S6. Predicted LIPSS periods Λ_{SPP} (a) and their possible uncertainty $\delta\Lambda_{\text{SPP}}$ due to dynamic changes of material optical properties in the course of laser irradiation (b). Λ_{SPP} and $\delta\Lambda_{\text{SPP}}$ are given in nm as a function of the real and imaginary parts of the dielectric permittivity ε at the interface with air / vacuum. In these estimating calculations, the fluctuations of the dielectric permittivity were assumed to be equal to $|\delta\varepsilon| = 5$. The data are presented for $\lambda = 1030\,\text{nm}$.

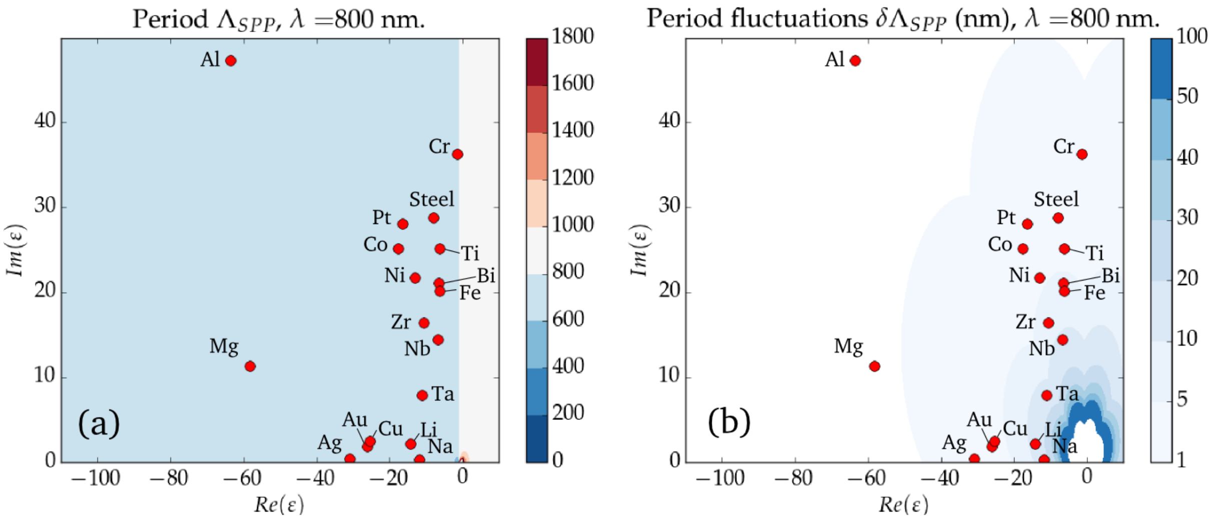

Figure S7. The same as in Fig. S6 for $\lambda = 800\,\text{nm}$. Note strong changes in the period, Λ_{SPP}, for materials whose dielectric permittivity is close to $0 + 0i$.

transient variations of light absorption periods on the surfaces of Ti, Fe, Bi, Ni, Co, Pt, Cu, Ag, Au and Mg are expected to be small, within 10 nm for the case of $-5 \leq \delta\varepsilon \leq 5$.

We recall that the spatial period Λ_{SPP} of the electromagnetic field on the metallic surfaces originated from the interference of the incident laser light with the excited SPP fields can be calculated from the complex-valued SPP wavenumber β via[6,20–22]

$$\Lambda_{\mathrm{SPP}} = \frac{2\pi}{\Re e\,(\beta)}.$$

For a general case of an interface between two media with dielectric functions ε_1 and ε_2, one can write

$$\Re e\,(\beta) = \frac{\omega}{2c}\sqrt{2\,\left[F_1^2 + F_2^2\right]^{1/2} + 2F_1}, \tag{3}$$

where

$$F_1 = \left(\varepsilon_1'\varepsilon_2' - \varepsilon_1''\varepsilon_2''\right)\left(\varepsilon_1' + \varepsilon_2'\right)F_0 + \left(\varepsilon_1''\varepsilon_2' + \varepsilon_1'\varepsilon_2''\right)\left(\varepsilon_1'' + \varepsilon_2''\right)F_0,$$

$$F_2 = \left(\varepsilon_1''\varepsilon_2' + \varepsilon_1'\varepsilon_2''\right)\left(\varepsilon_1' + \varepsilon_2'\right)F_0 - \left(\varepsilon_1'\varepsilon_2' - \varepsilon_1''\varepsilon_2''\right)\left(\varepsilon_1'' + \varepsilon_2''\right)F_0,$$

$$F_0 = \left[\left(\varepsilon_1' + \varepsilon_2'\right)^2 + \left(\varepsilon_1'' + \varepsilon_2''\right)^2\right]^{-1}.$$

Here symbols prime ($'$) and double prime ($''$) refer to the real and imaginary parts of the dielectric functions.

The LIPSS period uncertainty can be calculated as follows. Assuming irradiation by monochromatic laser light λ ($\delta\lambda/\lambda \ll 1$), the uncertainty of $\Lambda_{\mathrm{SPP}} \pm \delta\Lambda_{\mathrm{SPP}}$, is limited to

$$|\delta\Lambda_{\mathrm{SPP}}| \leq 2\pi\left|\frac{\delta\left[\Re e\,(\beta)\right]}{\Re e\,(\beta)^2}\right|$$

where the total differential of Eq. (3) gives the maximum error of

$$\delta\left[\Re e\,(\beta)\right] \leq \left|\frac{\partial\left[\Re e\,(\beta)\right]}{\partial\varepsilon_1'}\delta\varepsilon_1'\right| + \left|\frac{\partial\left[\Re e\,(\beta)\right]}{\partial\varepsilon''_1}\delta\varepsilon''_1\right| + \left|\frac{\partial\left[\Re e\,(\beta)\right]}{\partial\varepsilon'_2}\delta\varepsilon'_2\right| + \left|\frac{\partial\left[\Re e\,(\beta)\right]}{\partial\varepsilon''_2}\delta\varepsilon''_2\right|$$

Note that the real and imaginary parts of the dielectric function can fluctuate non-monotonously during the laser irradiation.[12] Figures S6(b) and S7(b) present the calculated uncertainties $\delta\Lambda_{\mathrm{SPP}}$ for metals irradiated

Interfaces	Sample thickness	L_{SPP} at 1030 nm Single / Multi	Optical penetration depth	SPP decay depth	Period Λ at 1030 nm Single / Multi
Air/Mo/SiO_2	300 nm	4.3 μm / 4.3 μm	18.2 nm	240 nm	1019 nm / 1019 nm
Air/Ti/SiO_2	300 nm	4.6 μm / 4.54 μm	15.7 nm	253 nm	1027 nm / 1027 nm

Table 2. Theoretical analysis of the effect of thin film thickness. Two models were compared: SPP model accounting for the thickness of the metal (named *Multilayer model*), and a *single* SPP model, assuming a semi-infinite bulk. Results clearly show that the metal films are optically thick.

in air ($\varepsilon_1 = 1$) at two wavelengths, 1030 and 800 nm respectively. It is demonstrated that the "transient" LIPSS period (or, strictly speaking, laser light absorption period[19]) can strongly vary for materials which exhibit a transient dielectric permittivity close to 0+0i (indicated by flower-like white region). Close to this particular point, temporal fluctuations of light absorption periodicity must be dramatic, leading, most probably, to a complete loss of LIPSS regularity. Out of this region, $\delta\Lambda_{\mathrm{SPP}}$ was found close to 10 nm for all metals of our study, assuming that uncertainty of ε is limited to $\delta\varepsilon = 5$. We mention that, in the cases of laser-irradiated semiconductor and dielectric materials, the transient excitation of electrons leads to a strong decrease of real part toward negative values and to an increase of the imaginary part by several units.[3,23]

Note that this simplified model can overestimate the LIPSS period uncertainty $\delta\Lambda_{\mathrm{SPP}}$. The more advanced physical model presented in the manuscript, which accounts for the transient change of the dielectric function $\varepsilon(t)$ upon laser irradiation, should yield in better predictions for both LIPSS periodicity and period uncertainties.

S7 Optical model of metallic films

A film of metal deposited on a substrate can occur to be non-fully optically thick. In particular, when considering excitation of surface plasmon polaritons (SPP), their properties can be affected by the interference between the exponential tails at the two sides of the film. Table 2 demonstrates that the prepared films of Ti and Mo samples (see Materials and Methods) can be considered as optically thick. For this purpose, two theoretical models were compared: (i) a classical SPP model[6,21] (ii) and a multilayer SPP model.[21,24] In the context of LIPSS, these models were applied in the case of femtosecond-laser-excited silicon immersed in water, to explain the reduction of the LIPSS period when thin films are irradiated.[25] Here we apply this model to the case of metallic films deposited on a quartz substrate to check the effect of film thickness on the SPP properties such as SPP period Λ, and SPP decay length L_{SPP}. At the irradiation

wavelength (1030 nm), both models give the same SPP optical properties, proving that the 300-nm-thick Mo and Ti samples can be considered as optically thick.

S8 Estimations of heat accumulation under the LIPSS formation on Ti and Mo films

Here on the example of titanium, we show that, between two successive pulses, the laser energy absorbed during a pulse diffuses from the irradiation spot in the irradiation regimes exploited in this manuscript.

First, one can estimate the maximum lattice temperature at the surface. Considering that electron-phonon themalization occurs at ∼10 ps after the laser pulse action and using data on the electron heat conductivity reported by Lin et al.[11], the thickness of the laser-affected layer can be evaluated by the end of electron-phonon equilibration. Similar estimations for titanium films irradiated by femtosecond laser pulses with the necessary parameters are reported by Goodfriend et al.[26] By applying this formalism to our conditions for titanium as an example, it can be shown that, by 10 ps after the laser pulse, the electron heat diffusivity transfers the heat to only 20-30 nm toward the film depth while the maximum temperature of the surface can reach or even exceed 10000 K. Thus, in our irradiation regimes, the LIPSS production is indeed performed in the regime of strong ablation of a thin external layer of titanium of the order of several tens of nanometers.

A substantial part of the heat is removed from the sample with the ablation products. We can assume that almost all molten material is ejected from the surface due to the phase explosion and the mechanical stresses already on the picosecond time scale.[27] The residual heat is then diffusing across the film and radially from the center of the irradiation spot. Propagation distance of the heat front between the pulses can be evaluated as $\Delta x \sim \sqrt{\chi t}$ where χ is the temperature diffusivity. For the interpulse time of $t \sim 1.6$ μs, we obtain for titanium the distance of ∼4 μm, close to the radius of the irradiation spot. Taking into account the cylindrical geometry of heat transfer in the metallic film, it is clear that the heat, after equalization across the film, is spread radially to much larger volume than that initially affected by the laser beam. Also, partially the heat is transferred into the low-heat-conducting dielectric substrate. Although a more exact dynamics of heat transfer can be clarified by numerical simulation, it can be concluded that, for only 2-4 subsequent pulses, heat accumulation cannot play an important role in our irradiation regimes. Also, it can be stated that the optical properties of the irradiated material (dielectric function) have to restore to $\sim 1\mu$s after the pulse action, hence, to the next laser pulse. However, arising wavelength-scaled

surface roughness due to generated LIPSS can influence the surface absorptivity.

Similar estimations can be done for molybdenum films though the estimation presented above gives the data typical for metals. It should be noted that the heat accumulation effect has to be an important element for the regimes of oxidation with thousands or even millions pulses coupling to the same spot on the surface, especially taking into account a reduced heat transfer in oxidized metals.[28]

S9 Effect of surface roughness

Roughness parameters	Ti	Mo	Steel	Cu	Al	Au
S_a (nm)	5.7	3.5	11.9	12.7	7.9	5.56
S_z (nm)	7.7	4.7	16.7	16.4	10.2	6.96

Table 3. Measurements of the surface roughness for each processed materials. S_a and S_z denote the arithmetic average height and the maximum height respectively.

Table 3 presents the results of AFM measurements of the surface roughness of all metal samples laser-processed in this study. As it is seen, the regularity is not directly connected with this surface parameter. Materials from two groups with high and poor regularity can have a similar roughness, compare Ti and Au as well as steel and Cu. Interesting is that the metal with smallest roughness, Mo, requires a higher overlapping for imprinting the highly-regular LIPSS (72.8% against 51.9% for Ti and steel), the aspect calling for further studies.

S10 Acknowledgment

The research of Y.L., N.M.B., and T.M. is co-financed by the European Regional Development Fund and the state budget of the Czech Republic (project BIATRI: CZ.02.1.01/0.0/0.0/15_003/0000445) and by the Ministry of Education, Youth and Sports (Programs NPU I-project no. LO1602, and Large Research Infrastructure-project no. LM2015086). T.J.-Y.D. acknowledges the support of Marie Skłodowska Curie Actions (MSCA) Individual Fellowship of the European Union's (EU) Horizon 2020 Program under grant agreement "QuantumLaP" No. 657424.

References

1. Imboden, M. & Bishop, D. Top-down nanomanufacturing. *Phys. Today* **67**, 45–50 (2014).

2. Bäuerle, D. *Laser Processing and Chemistry* (Springer-Verlag, 2011), 4th edn.

3. Derrien, T. J.-Y., Itina, T. E., Torres, R., Sarnet, T. & Sentis, M. Possible surface plasmon polariton excitation under femtosecond laser irradiation of silicon. *J. Appl. Phys.* **114**, 083104 (2013).

4. Ionin, A. *et al.* Sub-100 nm transverse gratings written by femtosecond laser pulses on a titanium surface. *Laser Phys. Lett.* **10**, 056004 (2013).

5. Bonse, J., Höhm, S., Rosenfeld, A. & Krüger, J. Sub-100-nm laser-induced periodic surface structures upon irradiation of titanium by Ti-sapphire femtosecond laser pulses in air. *Appl. Phys. A* **110**, 547–551 (2013).

6. Derrien, T. J.-Y., Krüger, J. & Bonse, J. Properties of surface plasmon polaritons on lossy materials: lifetimes, periods and excitation conditions. *J. Opt.* **18**, 115007 (2016).

7. Kaganov, M. I., Lifshitz, I. M. & Tanatarov, L. V. Relaxation between electrons and the crystalline lattice. *J. Exp. Theor. Phys.* **4**, 173 (1957).

8. Anisimov, S. I., Kapeliovich, B. L. & Perel'man, T. L. Electron emission from metal surfaces exposed to ultrashort laser pulses. *Sov. Phys. Technol. Phys.* **39**, 375–377 (1974).

9. Wellershoff, S.-S., Hohlfeld, J., Güdde, J. & Matthias, E. The role of electron-phonon coupling in femtosecond laser damage of metals. *Appl. Phys. A* **69**, S99–S107 (1999).

10. Qiu, T. Q. & Tien, C. L. Short-pulse laser heating on metals. *Int. J. Heat Mass Tran.* **35**, 719–726 (1992).

11. Lin, Z., Zhigilei, L. V. & Celli, V. Electron-phonon coupling and electron heat capacity of metals under conditions of strong electron-phonon nonequilibrium. *Physical Review B* **77**, 075133 (2008).

12. Kirkwood, S. E., Tsui, Y. Y., Fedosejevs, R., Brantov, A. V. & Bychenkov, V. Y. Experimental and theoretical study of absorption of femtosecond laser pulses in interaction with solid copper targets. *Phys. Rev. B* **79**, 144120 (2009).

13. Groeneveld, R. H. M., Sprik, R. & Lagendijk, A. Femtosecond spectroscopy of electron-electron and electron-phonon energy relaxation in Ag and Au. *Phys. Rev. B* **51**, 11433–11445 (1995).

14. Johnson, P. B. & Christy, R. W. Optical constants of the noble metals. *Phys. Rev. B* **6**, 4370–4379 (1972).

15. Godunov, S. K. & Ryabenkii, V. S. *Differences Schemes, An Introduction to the Underlying Theory* (Elsevier Science Publishers B.V., 1987).

16. Ordal, M. A., Bell, R. J., Alexander, R. W., Newquist, L. A. & Querry, M. R. Optical properties of Al, Fe, Ti, Ta, W, and Mo at submillimeter wavelengths. *Appl. Opt.* **27**, 1203–1209 (1988).

17. Panda, B. P. Electronic structure and equilibrium properties of hcp titanium and zirconium. *Pramana* **79**, 327–335 (2012).

18. Duma, I., Mel'nichuk, B. & Stasyuk, Z. Electrical conductivity and thermoelectric emf of nickel and molybdenum films. *Russian Phys. J.* **35**, 1123–1126 (1992).

19. Sipe, J. E., Young, J. F., Preston, J. & Driel, H. V. Laser-induced periodic surface structure. I. Theory. *Phys. Rev. B* **27**, 1141–1154 (1983).

20. Bell, R. J., Alexander, R. W., Parks, W. F. & Kovener, G. Surface excitations in absorbing media. *Opt. Commun.* **8**, 147–150 (1973).

21. Maier, S. A. *Plasmonics, Fundamentals and Applications* (Springer, 2007).

22. Ionin, A. *et al.* Femtosecond laser fabrication of sub-diffraction nanoripples on wet Al surface in multi-filamentation regime: High optical harmonics effects? *Appl. Surf. Sci.* **292**, 678–681 (2014).

23. Sokolowski-Tinten, K. & von der Linde, D. Generation of dense electron-hole plasmas in silicon. *Phys. Rev. B* **61**, 2643–2650 (2000).

24. Prade, B., Vinet, J. & Mysyrowicz, A. Guided optical waves in planar heterostructures with negative dielectric constant. *Phys. Rev. B* **44**, 13556 (1991).

25. Derrien, T. J.-Y. *et al.* Plasmonic formation mechanism of periodic 100-nm-structures upon femtosecond laser irradiation of silicon in water. *J. Appl. Phys.* **116**, 074902 (2014).

26. Goodfriend, N. T. *et al.* Laser pulse duration dependence of blister formation on back-radiated Ti thin films for BB-LIFT. *Appl. Phys. A* **122**, 154 (2016).

27. Gnilitskyi, I., Gruzdev, V., Bulgakova, N. M., Mocek, T. & Orazi, L. Mechanisms of high-regularity periodic structuring of silicon surface by sub-MHz repetition rate ultrashort laser pulses. *Appl. Phys. Lett.* **109**, 143101 (2016).

28. Öktem, B. *et al.* Nonlinear laser lithography for indefinitely large-area nanostructuring with femtosecond pulses. *Nat. Photon.* **7**, 897–901 (2013).
